# Insulin Resistance, Hyperandrogenism, and Its Associated Symptoms Are the Precipitating Factors for Depression in Women With Polycystic Ovarian Syndrome

**DOI:** 10.7759/cureus.18013

**Published:** 2021-09-16

**Authors:** Aarthi Ethirajulu, Almothana Alkasabera, Chike B Onyali, Comfort Anim-Koranteng, Hira E Shah, Nitin Bhawnani, Jihan A Mostafa

**Affiliations:** 1 Internal Medicine, California Institute of Behavioral Neurosciences & Psychology, Fairfield, USA; 2 General Medicine, California Institute of Behavioral Neurosciences & Psychology, Fairfield, USA; 3 Medicine, California Institute of Behavioral Neurosciences & Psychology, Fairfield, USA; 4 Psychiatry, California Institute of Behavioral Neurosciences & Psychology, Fairfield, USA

**Keywords:** polycystic ovary syndrome (pcos), depression, etiology, pathophysiology, treatment, insulin resistance, hyperandrogenism, obesity

## Abstract

Polycystic ovarian syndrome (PCOS) is a combination of many symptoms resulting from hormonal imbalance, metabolic syndromes, hyperandrogenism, and anovulation. This paper explores the various etiopathology and mechanisms causing depression in women with PCOS and how to prevent and treat PCOS-induced depression. Women with PCOS present with multiple symptoms such as acne, hirsutism, androgenic alopecia, obesity, menstrual irregularities, infertility, and mood disturbances like depression and anxiety. Depression is the most common psychological problem faced by women with PCOS. The various pathophysiological mechanisms that lead to depression are Insulin resistance, disturbance in the hypothalamic pituitary adrenal (HPA) axis, hyperandrogenism and its clinical presentation, obesity, and infertility. Lifestyle modifications such as dietary changes and weight loss play a significant role in preventing and managing PCOS-induced depression. Cognitive behavioral therapy (CBT) and lifestyle modification have shown to be effective measures for weight loss in obese women with PCOS. Antidepressants also play a part in treating PCOS-induced depression. Over the last decade, the number of cases of depression in women with PCOS has increased. This paper provides detailed data on the fundamental causes of depression in women with PCOS to facilitate a more straightforward treatment approach.

## Introduction and background

Etiology of depression in women with PCOS

The clear-cut etiology of polycystic ovarian syndrome (PCOS)-associated depression has not been postulated yet. PCOS is the most common endocrine disorder among women of the reproductive age group. These women present with reproductive, endocrine, metabolic, and psychiatric abnormalities [1]. Women with PCOS have an eight times higher prevalence of depression than women without PCOS [2]. A case-control study in Oman came up with results of the adjusted OR indicating an increased risk of depression (OR =1.10; 95% CI 0.50, 2.43) [3].

Further, increased activity of pro-inflammatory markers and activation of the immune system during stress related to PCOS is said to be one of the pathophysiologic factors causing depression [4]. This is induced by various factors such as obesity, infertility, acne, androgenic alopecia, and many other PCOS-associated symptoms.

The criterion for diagnosing PCOS is a triad that includes menstrual irregularities, imaging studies showing polycystic ovaries, and hyperandrogenism manifestations [5]. Some authors have hypothesized that PCOS symptoms, including obesity, infertility, and cutaneous stigmata of hyperandrogenism, such as hirsutism and acne, are linked to depression [6,7]. PCOS is a condition that affects women from a young age and its consequence extends throughout their lifetime. The management and treatment of PCOS depend on treating the most problematic symptom from the patient's perspective. More information must be obtained to identify the most disturbing symptoms so that treatment can be individualized according to the patients' needs [8]. The exact reasons for the increasing prevalence of depression in women with PCOS are yet to be explored [9]. In a qualitative study on the subjective experience of PCOS, women reported that they felt less feminine and attractive due to the symptoms associated with the disease, thereby mentioning PCOS as the “thief of womanhood” [10].

The motive of the present review is to know more about the etiology, pathophysiology of PCOS-induced depression and to know more about its prevention and treatment. Even though many studies and research have been conducted on this disease, there is no clear understanding of the depression caused in women with PCOS.

## Review

An overview of PCOS

PCOS is diagnosed when a group of symptoms consistently occur together as a result of hormonal imbalance such as obesity, anovulation leading to multiple cysts in the ovaries, infertility, increased peripheral estrogen that gets converted to androgen leading to hirsutism in young females, metabolic derangements like insulin resistance, and dyslipidemia leading to coronary artery disease [11]. It is one of the most common endocrine disorders affecting women in their reproductive age group (5-10%) and is one of the most common causes of female infertility [12].

In the past, studies were directing insulin resistance and hyperandrogenism as the underlying cause of PCOS-induced depression, as hyperandrogenism can lead to stigmatizing symptoms in women and decrease their quality of life, which in turn leads to a depressed mood [13]. A double-blinded randomized control trial (RCT) by Greenwood et al. published in 2018 concluded a strong association of insulin resistance to depression in women with PCOS [14]. Increased circulating testosterone levels have also been considered a biochemical factor for increased depression in PCOS [15]. Furthermore, dysregulation of the hypothalamic pituitary adrenal (HPA) axis plays a role in the cause of depression in PCOS [16]. A simple explanation is that there is impaired signaling in this axis that leads to decreased follicle stimulating hormone (FSH). This leads to anovulation, polycystic ovaries, and increased luteinizing hormone (LH), which increases the circulating androgens. This, in turn, gets peripherally converted to estrogen leading to suppression of gonadotropin-releasing hormone (GnRH) release from the hypothalamus in a negative feedback cycle. The etiology for PCOS-associated depression is not understood clearly. It is multifactorial.

The RCT by Greenwood et al. with 738 women with PCOS strongly suggests that insulin resistance is a causative factor for PCOS-associated depression [14]. In a univariate logistic regression analysis, the elevated value of homeostatic model assessment of insulin resistance (HOMA-IR) index was associated with a 2.3-fold increased odds of depression (OR 2.32, 95% CI 1.28-4.21, p<0.01). This association remained significant after controlling for age and BMI (adjusted OR (aOR) 2.23, 95% CI 1.11-4.46, p=0.02) and in a model including additional potential confounders (aOR 2.03, CI 1.00-4.16, p=0.05) when compared to a cross-sectional study that concludes that body image distress (BID) causes depression in women with PCOS [17]. In the above comparison, the RCT is more reliable than the univariate logistic regression analysis. Therefore, insulin resistance is considered the primary cause of depression in women with PCOS.

A meta-analysis by Coony et al. on depressive symptoms included 18 studies [2]. The results were as follows: women with PCOS had increased odds of any depressive symptoms (OR: 3.78; 95% CI: 3.03-4.72; 18 studies) and of moderate/severe depressive symptoms (OR: 4.18; 95% CI: 2.68-6.52; 11 studies), which supports that PCOS is associated with depressive symptoms. Another cross-sectional study in India with a sample size of 70, which used the Hamilton rating scale for depression, concluded that the prevalence of depression was 25.7% in women with PCOS [18]. Comparing these studies, although both studies conclude that PCOS is associated with depression, we find that the study by Cooney is more reliable.

The pathophysiology behind development of depression in PCOS

**Figure 1:**
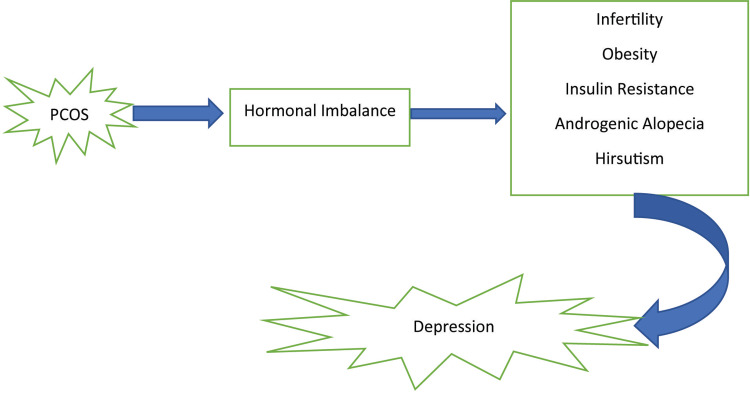
Multifactorial causes of PCOS-associated depression PCOS: Polycystic ovarian syndrome

The clinical manifestations of PCOS, especially obesity and infertility, are associated with depression in women with PCOS compared to women without PCOS experiencing similar symptoms [19]. Other factors leading to depression in PCOS are insulin resistance, increased circulating testosterone, hyperandrogenism, and HPA dysregulation [14,15,13,16]. Inflammation of the hypothalamus has been associated with the clinical, hormonal, and metabolic changes in PCOS [1]. However, as discussed earlier, an RCT by Greenwood et al. [14] concluded an independent association between insulin resistance and depression in PCOS. Therefore, the cause of depression in PCOS is multifactorial, as seen in Figure 1.

Although many different mechanisms have been proved to cause PCOS-associated depression, the approach should be catered to a specific underlying cause in a patient-specific manner. Therefore, the approach and treatment will vary according to the patient.

How to diagnose PCOS in a woman?

The diagnostic criteria include the following: chronic oligo or anovulation in women where serum FSH and LH should be tested, presence of numerous cysts in the ovaries in ultrasound examination, presence of elevated androgen levels [20]. PCOS is a condition that requires a multidisciplinary treatment strategy, as it is a heterogeneous condition presenting with reproductive, endocrine, metabolic, and psychiatric abnormalities.

Physical Examination Findings in Women With PCOS

**Figure 2:**
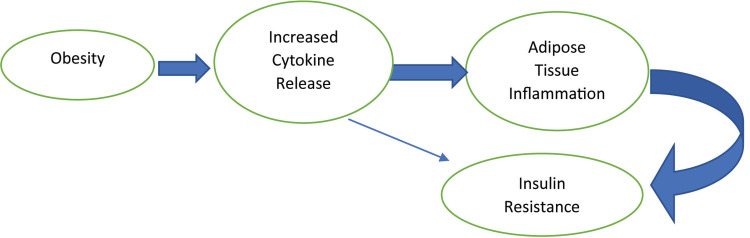
The overview of obesity leading to insulin resistance

Women with PCOS are diagnosed clinically based on the signs and symptoms of hyperandrogenism such as acne, increased facial hair growth, and androgenic alopecia. Previous studies show that 40-60% of women with PCOS are obese [19]. Being obese is one of the common factors causing depression in women with PCOS compared to women without PCOS [21]. Obesity, in turn, causes metabolic abnormalities such as insulin resistance (type-2 diabetes mellitus), as shown in Figure 2.

Diagnosing depression in women with PCOS is a crucial step that should be done early in the approach, as multiple studies support PCOS is associated with depression such as this comprehensive meta-analysis that shows that women with PCOS have four times the odds of depressive symptoms than women without PCOS [2]. In addition, another population-based cohort study by Greenwood et al. concluded that women with PCOS have higher depression scores during their lifetime [22]. The scoring was done using the Center for Epidemiologic Studies-Depression (CES-D) scoring system.

The diagnosis of PCOS is made using the three well-known criteria: anovulation/menstrual irregularities, signs and symptoms of hyperandrogenism along with elevated LH, circulating testosterone and decreased FSH in a hormonal assay, and observation of numerous cysts in bilateral ovaries in ultrasonogram.

Treatment of PCOS and PCOS-associated depression

The treatment for PCOS is individualized according to the patient’s presentation. Lifestyle changes with diet, exercise, and weight loss are considered the first line of treatment for women with PCOS who are obese or overweight. It reduces the BMI, overall body weight, waist to hip ratio, insulin resistance, and hyperandrogenemia followed by pharmacological management [23]. The depressive symptoms associated with BID may improve with lifestyle modifications [17]. In an RCT, weight loss of 10% and oral contraceptive pills (OCP), 20 μg ethinyl estradiol/1 mg norethindrone acetate every day, in combination was shown to decrease depressive symptoms in PCOS significantly [24]. Combined use of 50,000 IU vitamin D every two weeks and probiotic capsule containing four viable strains: *Lactobacillus acidophilus*, *Bifidobacterium bifidum*, *Lactobacillus reuteri*, and *Lactobacillus fermentum* (2 × 109 CFU/g each) for 12 weeks have been shown to improve PCOS-associated mental health [25]. For PCOS women with fertility issues, infertility management and reproductive techniques are used but are more stressful [25]. CBT in combination with lifestyle changes leads to faster and effective weight reduction and improvement in the quality of life in women with PCOS who are obese or overweight, which in turn reduces depression, according to Cooney et al. [26]. Pharmacological management of PCOS is done using the following medications: metformin for insulin resistance, spironolactone for hirsutism and insulin resistance, OCP for menstrual regulation, and the use of antidepressants like bupropion, dopamine and norepinephrine reuptake inhibitors, and naltrexone for PCOS-associated depression [27].

The treatment of PCOS needs a multidisciplinary approach. The mainstay of treatment is diet change and weight loss, which improves all the other symptoms associated with PCOS; when lifestyle modification fails, pharmacological agents like OCP, metformin, and other agents are used. But a combination of CBT, lifestyle management, and medication is shown to improve the quality of life in women with PCOS. 

An RCT by Dokras et al. with a study number of 132 women with PCOS were treated with lifestyle modification, OCP, and a combination of both for 16 weeks, and the result of the study shows that the use of OCP and lifestyle modification improved the overall quality of life in these women [24]. At baseline, 24.4% of subjects had depression or anxiety symptoms, and 6.8% were already on medication. Over the study period, the prevalence of depression, as assessed by positive screens on the Primary Care Evaluation of Mental Disorders (Prime-MD) and/or use of medications, decreased from 13.3 to 4.4% in the OCP group (OR, 0.30; 95% CI, 0.09, 0.99; P < .05) and from 22.7 to 15.9% in the lifestyle modification group (OR, 0.64; 95% CI, 0.34, 1.22; P = .17). The prevalence of depression did not change in the combined group (11.6 vs 11.9%). This study is superior when compared to the RCT by Cooney et al. [26] in which a total of 20 women were randomized to CBT and lifestyle modification and 13 to lifestyle modification. However, only seven in the first group and eight in the second group completed the whole RCT of weekly CBT for eight weeks and lifestyle modification of 16 weeks; they concluded that CBT combined with lifestyle modification resulted in weight loss and improved quality of life when compared to lifestyle modification alone. The CBT+lifestyle modification group lost more weekly weight (−0.35 kg/week vs. −0.16 kg/week) compared with the lifestyle modification group. Overall, the CBT + lifestyle modification group lost 3.2 kg versus 1.8 kg for the lifestyle modification group. The CBT+lifestyle modification group had greater improvement in the Polycystic Ovary Syndrome Health-Related Quality of Life Questionnaire (PCOSQ) at eight weeks (+3.7 vs. +1.2 points). This study's limitation is the selection of subjects and the minimal n=15. Therefore the RCT by Dokras et al. [24] is more reliable when compared to that by Cooney et al. [26]. While comparing both studies, RCT study with lifestyle modification and OCP is more significant and reliable.

Measures to prevent depression in PCOS

Weight loss and diet changes along with OCP play a key role in preventing complications in PCOS such as hyperandrogenism-related signs and symptoms and metabolic complications like type-2 diabetes mellitus [24]. In a study, Basirat et al. concluded that women with PCOS are more stressed when it comes to infertility issues and their management and that they need special attention and psychosocial care compared to normal women with infertility [28]. Along with lifestyle modification, CBT hastens weight loss in obese women with PCOS [26]. Furthermore, an RCT by Jiskoot et al. shows that PCOS-associated depression reduced with lifestyle modifications compared to care as usual [29].

Lifestyle modification is the key to prevent PCOS-associated complications and PCOS-associated depression. Women with PCOS must be provided a higher level of psychosocial care than those without PCOS, as PCOS is associated with depression.

PCOS-associated complications

PCOS leads to numerous complications, such as decreased cognitive functioning compared to women without PCOS [29]. Some of the other complications associated with PCOS are related to our topic of interest i.e PCOS-associated depression and other psychiatric conditions like anxiety and borderline personality disorder (BPD) [13]. PCOS also leads to metabolic complications such as type-2 diabetes mellitus, obesity, and cardiovascular disease [11]. Endometrial carcinoma is another complication in women with PCOS.

The complications related to PCOS lead to stress and depression. Hormonal imbalance and changes lead to most of the symptoms that rob the sense of womanhood from these women and also increase the risk of endometrial cancer.

Limitations

Almost all the references were from the studies conducted in the last fifteen years. Although several references were of high standards from RCTs, data was also collected from a few cross-sectional studies.

## Conclusions

PCOS is an endocrinological condition that affects many women in their reproductive age group. The presenting features vary from one woman to another. Depression is one of the most common mood disorders seen in women with PCOS. The exact mechanism causing depression in these women is not clear and is considered multifactorial such as inflammation of the hypothalamus, insulin resistance, and clinical features associated with hyperandrogenism. This paper provides a detailed explanation of the various causes of depression in women with PCOS and compiles important relevant information from several previous studies done on this topic.

A lot more is yet to be discovered regarding the pathophysiology behind PCOS-induced depression. Therefore, in the future, more RCTs should be conducted with large number of patients to know the exact mechanism of depression in PCOS, making it easier to treat these women who suffer from PCOS-induced depression. At present, the treatment of PCOS-induced depression is the same as depression from any other cause. The main focus should be the prevention of signs and symptoms of PCOS that cause depression. The preventative measures are weight loss and dietary changes along with CBT.
